# Responding to Moral Challenges in Clinical Practice: A Qualitative Assessment of Clinical Ethics Support Needs at Three Tanzanian Hospitals

**DOI:** 10.1007/s10730-025-09547-8

**Published:** 2025-04-16

**Authors:** Shija Kevin Kuhumba, Trygve Johannes Lereim Sævareid, Nandera Ernest Mhando, Bert A.C. Molewijk

**Affiliations:** 1 Centre for Medical Ethics, Faculty of Medicine, University of Oslo, Oslo, Norway; 2 Department of Philosophy and Religious Studies, College of Humanities, University of Dar Es Salaam, Dar Es Salaam, Tanzania; 3 Department of Health and Nursing Science, University of Agder, Kristiansand, Norway; 4 The Social Welfare Department in the Ministry of Community Development, Gender, Women, and Special Groups, Dodoma, Tanzania; 5 Department of Sociology and Anthropology, University of Dar Es Salaam, Dar Es Salaam, Tanzania; 6 Department Ethics, Law and Humanities, Amsterdam UMC (University Medical Centres), Amsterdam, the Netherlands

**Keywords:** Moral challenges, Clinical ethics support, Clinical ethics committee, Healthcare professionals, Clinical practice, Tanzania

## Abstract

Healthcare professionals (HCPs) encounter various moral challenges in clinical practice. In various countries, clinical ethics support (CES) services are developed to support HCPs. One of these CES services is clinical ethics committees (CECs): they address moral challenges faced in healthcare settings and offer support for HCPs. However, in Tanzania, CECs have not yet been implemented. For implementation purposes, greater knowledge about how healthcare professionals navigate and respond to moral challenges, their understanding of CECs, and what they perceive as key needs for implementing CECs in hospitals, are valuable. This study explores HCPs’ ways of dealing with their moral challenges at the moment and identifies key needs for establishing CECs in Tanzanian healthcare settings in the near future. The findings show that various implicit ways have been acknowledged as being useful in addressing moral challenges (e.g., regular meetings, family conferences, social welfare units, hospital procedures and guidelines, as well as consulting legal and management units). In addition, HCPs reported that a necessity exists for implementing more formal and systematic modalities to address moral challenges in clinical settings. The research in this paper has served as a preparation for establishing the first CEC in Tanzanian healthcare settings.

**Supplementary Information:**

The online version contains supplementary material available at 10.1007/s10730-025-09547-8.

## Introduction

Healthcare professionals encounter various types of moral challenges in their clinical practice, contributing to the complexity of decision-making (Blanco Portillo et al., 2021; Ong et al., 2020). Examples of situations that may lead to moral challenges are disagreement among family members, scarcity of medical resources, disagreement among healthcare professionals, conflicts between professional judgment, religious convictions and alternative treatments, challenges related to decision-making, challenges related to communication and truth-telling as well as dispensing medicines during pandemics such as COVID-19 (Blanco Portillo et al., 2021; Cox, 2021; DuVal et al., 2004; Hurst et al., 2007; Kuhumba et al., 2024; Miljeteig et al., 2019). Not recognizing or ignoring *moral* challenges and not being aware of how to constructively deal with them might be detrimental to patients and relatives and can also affect healthcare professionals and hospital management in negative ways. Ultimately it might affect the quality of care (Hem et al., 2018). Several studies have indicated the usefulness of CES services in dealing with complex ethical issues in clinical settings (Cohn et al., 2007; Magelssen et al., 2020a, 2020b), improving the moral and professionals’ competency of HCPs (Hem et al., 2018; Molewijk et al., 2008a, 2008b), as well as fostering a spirit of dialogue in addressing moral challenges (Janssens et al., 2015).

Various CES services have been developed to assist in recognizing, reflecting upon and dealing with moral challenges. CES services are usually offered via the use of protocols or thematic ethics support tools (Gerritse et al., 2023; Ligtenberg et al., 2024), a CEC (Førde & Pedersen, 2011; Magelssen et al., 2021), clinical ethics consultation,moral case deliberations (MCD) (Jakobsen et al., 2024; Rasoal, 2018; Rasoal et al., 2016), and ethics rounds (Molewijk et al., 2008a, 2008b; Stolper et al., 2012).

In most Sub-Saharan African countries, CES services have not been systematically implemented (Aboud et al., 2018; Moodley et al., 2021). Approaches that have been used include clinical meetings, consulting colleagues, informal discussions among colleagues, and discussions in various types of regular meetings at the unit/ department (Nanyonga et al., 2024). Yet it is unclear how these approaches focus on the moral dimension of the situation, the central moral questions addressed, and the moral reasoning used in addressing moral challenges.

Knowledge about how healthcare professionals deal with moral challenges in clinical practice in Sub-Saharan Africa is sparse (Dzi et al., 2024; Ewuoso, 2019, 2020; Nanyonga et al., 2024). Similarly, little is known about how healthcare professionals in Tanzania address moral challenges in clinical practice and the key needs for establishing CECs in Tanzanian healthcare settings. Understanding how healthcare professionals currently handle moral challenges and identifying their key needs for implementing a CEC is crucial. Therefore, this paper addresses three questions 1) How do Tanzanian healthcare professionals handle moral challenges? 2) What is their understanding of a CEC and the availability of CECs in hospitals? And 3) what key needs do they identify for the implementation of CECs in Tanzanian healthcare settings?

## Methods

This study is part of a previous study (Kuhumba et al., 2024) within the Enhancing Ethics and Integrity in Medical Research and Clinical Practice (ETHIMED) project. The project is financed by The Norwegian Directorate for Higher Education and Skills, through the Norwegian Partnership Programme for Global Academic Cooperation (NORPART) NORPART-2021/10586. ETHIMED project is a collaborative venture between the University of Oslo through Centre for Medical Ethics, the University of Dar es Salaam through the Department of Philosophy and Religious Studies, and the University of Rwanda through the College of Medicine and Health Sciences. In Tanzania, the project has focused on capacity building in clinical ethics support for healthcare professionals, research on clinical ethical issues, and establishing the first CEC in Tanzania at Mbeya Zonal Referral Hospital (MZRH).

### Design

We employed a qualitative research method with an explorative study design. It was a sound scientific method to explore how healthcare professionals respond to moral challenges and their key needs for establishing CECs in Tanzanian healthcare settings. The qualitative research method was selected because of its richness in exploring experiences, practices, and phenomena from sociocultural perspectives (Moen & Middelthon, 2015). We used the standards for reporting qualitative research (SRQR) as the checklist for reporting qualitative studies (O’Brien et al., 2014).

### Organizational Structure of Tanzania’s Health System

Tanzania is a low-middle-income country in Sub-Saharan Africa with approximately 61.8 million inhabitants (United Republic of Tanzania, 2023a). The health system structure comprises of public health sector, the private health sector which was expanded by the law which allowed private actors other than the Faith Based Organisations (FBOs) to own private hospitals in 1991, and traditional health and alternative health services (Temba, 2023).

**Fig. 1:**
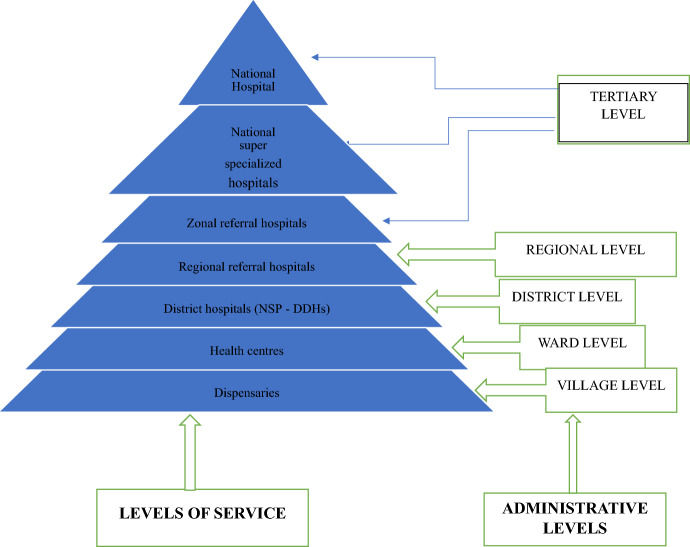
Structure of the healthcare system in Tanzania

The Tanzania health system has a mixture of both public and private health facilities providing different types of services depending on the level of administration (see Fig. 1). At the bottom, there are dispensaries that are found in every village and above this level there are health centers found at the ward level.

Dispensaries are healthcare facilities located at the village level, serving as the most basic unit within the community. They provide outpatient services, maternal and child healthcare, as well as community health services to their local areas. Each dispensary typically serves a population of 6,000 to 10,000 people and oversees all village health posts within its jurisdiction (United Republic of Tanzania—Ministry of Health, 2023a; United Republic of Tanzania, 2023b). The next level of healthcare facilities are health centers, which cater to a larger population of about 50,000 people within an administrative division known as a ward. Health centers offer a wider range of services, including emergency care, prenatal and obstetric care, and minor surgeries.

At the district level, there are district (Council) hospitals and designated district hospitals. Designated district hospitals are owned by private entities and subsidized by the government. The district hospitals provide services such as emergency care, major and minor surgery, delivery services, X-ray, and ultrasound services. Multiple districts are grouped into regions, each with a regional hospital. Regional hospitals provide specialized medical care such as general surgery, cardiology, nephrology, and gynaecology.

The tertiary level comprises zonal referral hospitals, national super specialized hospitals, and one national hospital. They provide advanced medical care, such as radiology services, CT scan, intensive care services, and ultrasound, and also functions as a teaching hospital for medical, paramedical, and nursing schools.

The Tanzanian health system is financed mainly through public sources (taxes and donor contributions), external sources (grants and loans) and private sources (out-of-pocket payments and insurance premiums). The main health insurance schemes in Tanzania are the National Health Insurance Fund (NHIF), the Social Health Insurance Benefits (SHIB) and the Improved Community Health Fund (iCHF) (Maluka et al., 2018; Kagaigai et al., 2021, 2024). Population coverage with health insurance has remained relatively low estimated at 15.3% of the total population (United Republic of Tanzania, 2023c). This indicates that the majority of the population rely on out-of-pocket [OOP] payments that expose them to catastrophic health expenditures (Kagaigai et al., 2023; Osoro et al., 2024). To address major health financing gaps, the Tanzania government has recently drafted a Universal Health Insurance UHI Act [2023] (United Republic of Tanzania, 2023d). Under the new law, all citizens must enrol in a health insurance plan, either public or private. A government fund will cover the cost of health insurance for those who are unable to afford medical costs out of their pocket (Buguzi, 2024).

### Study Participants

Study participants were recruited from three tertiary hospitals in Tanzania. These hospitals were purposely selected because they serve patients with more complex and severe cases which often raise moral issues. The study participants were recruited from intensive care units (ICU), emergency and internal medicine departments. Before data collection, the first author engaged in preliminary discussions with various Tanzanian healthcare professionals to identify specific departments for the study. Through these discussions, three specific departments were identified. The reason for consulting these healthcare professionals was that none of the authors had working experience in Tanzanian healthcare settings.

### Data Collection

All authors collaboratively developed the semi-structured interview guide (see Appendix 1). This collaboration ensured that the guide was comprehensive and aligned with the study’s research questions, rather than reflecting on a single perspective. The interview guide developed for this study ensured structured and consistent data collection, thereby mitigating potential researcher bias in several ways. First, it minimized the likelihood of the researcher engaging in unintentional conversations that might reflect personal perspectives and interests. Second, it established a framework that reduced the researcher’s influence on responses that could align with their expectations. Third, it maintained a focused and consistent examination of the core themes of the study, thereby offering a basis for systematic comparison of the collected data.

The data was collected from June to November 2023. The first round of interviews took place between June and September 2023. During this period, the first author interviewed 36 participants at their workplaces, with each interview lasting an average of 40 min. These interviews were audio-recorded. In November 2023, the first author conducted two additional interviews via phone calls with individuals holding key responsibilities at one of the study sites. These interviews were necessary to clarify research questions about how their hospital structures address moral challenges in clinical settings. The reasons for conducting these additional interviews were twofold. First, we aimed to gain deeper insights into how moral challenges in clinical practice are addressed, and to identify key needs for establishing a CEC, based on the analysis of the first 36 interviews. Second, these two additional interviews were conducted with healthcare practitioners from the selected departments who held key positions within the hospital structure and were directly involved in providing quality care. We believed that their perspectives on addressing moral challenges and their insights on key needs for establishing a CEC would significantly enrich the findings of this study. Unlike the initial interviews, these two were not audio-recorded; instead, the first author took notes, which were then integrated into the transcribed data set.

Furthermore, two of the initial 36 study participants were interviewed a second time via phone. These follow-up interviews were not audio-recorded; instead, the first author took notes during the conversations. The primary purpose of these follow-up interviews was to gather more information on how existing hospital structures, such as medical ethics units affiliated with the studied tertiary hospitals, assist healthcare professionals in addressing moral challenges in clinical settings.

All interviews were conducted in Swahili. Upon obtaining informed consent, the first author recorded the initial interviews. Only the 36 audio-recorded interviews were transcribed verbatim and translated from Swahili to English, allowing two authors unfamiliar with Swahili to access the data set, join in the analysis of the transcripts, and facilitating the drafting of the text about the research findings. To ensure anonymity, participant codes were used in the transcripts and field notes.

### Data Analysis

Reflexive thematic analysis (TA) was employed to analyze the collected data. Reflexive TA is a qualitative analysis framework developed by Braun & Clarke (2006; see also Braun & Clarke, 2013, 2021). Reflexive TA is a method for identifying, analyzing, and reporting patterns (themes) within data (Braun & Clarke, 2013). The iterative inductive data analysis entailed six stages. In the first stage, the first author repeatedly reviewed the data to become thoroughly familiar with the study results. Subsequently, the first author created a summary of the key findings. The other authors reviewed and commented on this summary, reflecting on the meaning and representativeness of these initial findings. Through this collaborative process, the researchers reached a coherent understanding of the results.

In the second stage, the first author created initial codes after familiarizing himself with the data, and the other authors reviewed and provided feedback on the created codes. In the third stage, overarching themes were formed by combining the initial created codes, created in the second stage. In the fourth stage, the themes created were thoroughly reviewed by the second and last authors. At this stage, we engaged in a rigorous process to ensure that the themes created represent the study findings. Also, at this stage, the first author reviewed the entire data set to check that the created themes reflect the meanings evident in the data set as a whole. In the fifth stage, the themes were re-defined and named. At this stage, we conducted a detailed analysis of each theme to capture the key aspects of the data. We identified sub-themes to provide a nuanced understanding of the data findings. Also, we selected vivid examples from the data to illustrate each theme. In the sixth stage, the first author drafted a preliminary report that included the introduction, research methods, findings, discussion, and conclusion. This preliminary report was then reviewed by the other authors, followed by discussions and further refinement until it was ready for final submission. In the analysis, we used NVivo 12 software in data coding and identifying recurring themes and subthemes. The interview transcripts were imported into the NVivo 12 software. Finally, the first author developed a table (see Appendix 2) indicating themes, sub-themes, codes, and illustrative quotes, which other authors reviewed.

### Backgrounds of the Researchers

Since our analysis is shaped by our professional and academic backgrounds, skills, experiences, and social positioning as researchers, we briefly pay attention to this. Authors have diversified academic and professional backgrounds. Two authors (TJLS and BM), have academic training and clinical experience in nursing. The two other authors have academic backgrounds in, respectively, sociology and social anthropology (NEM), and philosophy and ethics (SKK). The interdisciplinary nature of the research team has been significant in analysing data, developing themes and developing the paper from multidisciplinary perspectives. In terms of social positioning, two authors are from Northern Europe (TJLS and BM), while two are from Tanzania (SKK and NEM). In addition, three authors have been involved in clinical ethics (support) training (TJLS, BM, and SKK), and establishing the first CEC at one of the sites studied. None of the authors are working as healthcare practitioners in the Tanzanian healthcare setting.

### Ethics Considerations

The study was approved by various research ethics agencies, including the National Health Research Ethics Committee in Tanzania (NIMR/HQ/R.8a/VOL.IX/4237), relevant regional research ethics committees (RECs) and Independent Review Boards (IRB) at the selected hospitals, as well as the University of Dar es Salaam’s Research Ethics Committee (UDSM-REC). In addition, the Norwegian Data Protection Services for Research (Sikt) has received notification about the study and approved it (Ref.nr. 631,106). The study participants were informed about the fact that their participation in the study is voluntary and that they could withdraw from participation in the study at any time without giving reasons. Participants received detailed information about the study and its purpose, both orally by the first author as part of the introduction during the interview session for each study participant. After explaining the intention to audiotape the initial interviews, treating data confidentially, removing their names from the transcripts, and clarifying their right to withdraw from the study at any time, the study participants signed the informed consent form. For the interviews via phone calls, consent was obtained orally from the study participants.

## Findings

### Participants

Study participants were identified through the first author contacting the heads of selected departments. In each hospital studied, we had a gatekeeper familiar with the site who introduced the first author to the heads of departments for this study. After the first author presented the scope of the study, the department heads provided the first author with contact information for participants who met the inclusion criteria. Then, the first author contacted directly the study participants to seek an appointment for the interviews. A total of 38 participants were recruited. Physicians and nurses from the selected departments with more than three years of experience were included in the study. (Table 1)

**Table 1:** shows the sociodemographic characteristics of the study participants

Characteristic	No	Frequency
Gender		
Female	20	52.7%
Male	18	47.3%
Profession		
Nurses	21	55.3%
Physicians	17	44.7%
Sector		
Government hospitals	28	73.7%
Private owned hospital with government support	10	26.3%

We developed three overall thematic categories from the data: 1) reported approaches used in handling moral challenges in healthcare settings; 2) awareness and the status of CECs in healthcare settings; and 3) perceived key needs for establishing CECs.

### Theme 1: Reported Approaches Used to Handle Moral Challenges

When asked about approaches used in dealing with moral challenges in their healthcare settings five subthemes emerged out of the data analysis: (1) meetings within a team of healthcare professionals, (2) using the family conference, (3) involvement of the social welfare unit, (4) use of hospital procedures and guidelines in handling morally difficult decisions and (5) consulting management and legal units.

#### Subtheme 1: Meetings within a Team of Healthcare Professionals

Some healthcare professionals reported that moral challenges are discussed through various types of meetings at the department and unit levels. Meetings include formal departmental meetings, debriefing meetings, formal gatherings, morning meetings, and meetings between treatment teams to discuss the patient’s treatment plans. Supervisory sessions and mentorship programs for senior and junior healthcare professionals are included. However, meetings are not structured to systematically discuss moral challenges due to a lack of formal ethics support services: “… *it is mostly business as usual, so there is nothing new. I believe it is because we lack an effective system to track each case….*” (#22, female nurse, Hospital B). In addition, another participant mentioned that “*the existing structure investigates misconduct more frequently. We don’t have a formal system that performs an advisory role on moral dilemmas and offers training on clinical ethics*” (#10, male physician, hospital A).

Meetings are used to discuss moral challenges in clinical practice: “*We discuss moral challenges encountered in clinical settings*. *We learn from them, and how to address them if they arise again”* (#20, male nurse, hospital B). Moreover, at the departmental level, ethical issues are raised during the departmental meeting: “*There is also a departmental management meeting every Wednesday…. So, we discuss these things quite extensively*” (#5, male nurse, hospital A).

In addition, moral challenges encountered in clinical practice are discussed during supervisory meetings. One of the participants noted:

*There is usually a morning report in these meetings, where the supervisor discusses everything related to clinical practice on that particular day. Any dilemma that has arisen is discussed during the meeting…. These meetings are conducted at the departmental level.* (#12, male nurse, hospital A)

Another participant reported that meetings are used as education sessions for junior and newly recruited healthcare professionals: *“We educate and remind each other because some of the staff are newcomers, so they need to understand how to handle these challenges, and if they can’t, they know who to seek guidance from”* (#17, female nurse, hospital B).

However, it was reported that regular meetings are not multidisciplinary. One female nurse said: “*In such meetings, experts in medical ethics, bioethics and ethics are not invited to assist in analyzing the case….”* (#9, female nurse, hospital A).

The participants also reported that mentorship programmes are used in dealing with distressing situations, particularly in the ICU, and emergency department.

*We hold weekly mentorship sessions to equip mostly junior medical staff with skills in handling moral distress in the ICU environment. Such sessions aim to strengthen healthcare practitioners and be morally resilient to ethical challenges encountered in the healthcare setting.* (#15, male physician, hospital A)

*Our department has a mechanism called mentorship families, where every junior doctor is assigned a senior specialist mentor. Your mentor is readily available, especially when you need to discuss moral challenges you encounter in clinical practice*. (#8, female physician, hospital A)

It was indicated that in some circumstances, the team of healthcare professionals was used to decide the best course of action in handling some clinical cases, especially in minimizing and withdrawing medical care and prioritizing patients in clinical practice.

*If the cancer patient is already on the ventilator, you cannot remove them just because another patient has arrived… In such situations, a team of healthcare professionals and a physician on duty decide who will benefit from the therapy based on the scarce medical resources available at the hospital unit.* (#10, male physician, hospital A)

#### Subtheme 2: Making use of the Family Conference

Participants indicated some moral challenges are handled through the involvement of the family members. Family conferences are mostly used in the ICU. Participants noted the occurrence of regular family conferences but did not elaborate on how these conferences are used to address moral challenges. Additionally, they did not specify whether these family conferences are held before or after moral challenges arise in clinical settings. The study findings indicate that family conferences can take the form of both formal and informal meetings. Family conferences can range from casual discussions between a healthcare professional and a family member to more structured meetings involving the patient, their relatives or next of kin, and healthcare professionals. These healthcare professionals may include the nurse responsible for the patient, the attending physician, the block manager, and the department head (#4, male nurse, hospital A).

It was reported that family conferences serve as platforms for healthcare professionals to communicate with family members about the patients’ complex health conditions. One participant mentioned that in case of moral uncertainties arising in clinical practices such as dealing with a patient with brain death. They convene an emergency family conference to explain the situation and involve family members in decision-making (#28, female nurse, hospital C).

Another participant mentioned that:

*We have family conferences because when a patient is in the ICU, there is usually a lot of confusion and uncertainty about what has to be done. So, we involve them at every step. We call the family members and explain why the patient was brought to the ICU and the treatment plans. We also listen to their preferences, such as their desire for spiritual support services for their loved ones, which we accommodate*.” (#19, male nurse, hospital B)

Also, participants reported that family conferences are used to offer education to family members with the intention of assisting them in participating fully in the decision-making processes and deliberating upon moral challenges encountered in clinical practice.

*The critical issue is usually to inform the relatives because the patient belongs to them, and they need to make decisions. It is their autonomy so a patient might need a specific treatment, but the family might disagree. Your role is to educate them. If they refuse, you document their refusal so that later, they don’t turn around and accuse you of terminating the patient’s treatment*. (#10, male physician, hospital A)

Participants indicated situations where healthcare professionals manage moral challenges associated with refusal of treatment through educational sessions with patients and family members. One male physician noted: “*My job is to educate and make them understand the treatment’s benefits and the consequences of declining the therapy*…” (#6, male physician, Hospital A).

Another participant mentioned engaging patients and family members through counselling sessions upon refusal of treatment.

*We … provide counselling. It is challenging to withhold a patient from treatment just because relatives have requested it, so we make a significant effort to provide counselling. So far, counselling has been the primary approach to overcoming moral challenges related to the disagreement between family members and healthcare professionals on patient care.* (#34, female physician, hospital C)

Counselling sessions aid a team of healthcare practitioners and parents in making decisions based on the patient’s health condition:

*After the counselling process, a team of healthcare practitioners and parents decides to de-escalate or minimize care to the patient and allow the natural course to occur. It isn’t easy to make such decisions, but we must make them… we involve parents in making such medical decisions.* (#15, male physician, hospital A)

#### Subtheme 3: Involvement of a Social Welfare Unit

Participants reported that the social welfare units in the hospital setting assist them in managing moral challenges related to patients’ and families’ inability to cover medical costs since some patients do not have medical insurance and thus are inclined to out-of-pocket payments. One of the participants said:

*We use social welfare personnel, especially in social and economic issues, especially when a person feels that medical treatment is too expensive and can’t afford it. The social welfare team provides counselling, and the patient continues to receive treatment while they assess the family’s ability to contribute to the medical expenses. They visit the patient’s home, interact with the local government authorities, and evaluate the situation to confirm that the patient is being treated under the exemption. * (#23, female nurse, hospital B)

Social welfare officers could assist in handling a moral challenge related to refusing treatment by the patient’s family members.

*Sometimes, patients are required to go to the operating theatre, but their family refuses. The patient might be an adult but unable to make decisions for themselves. At the same time, we continue to persuade the family by involving social welfare personnel….* (#21, male nurse, hospital B)

#### Subtheme 4: Use of Hospital Procedures and Guidelines in Handling Morally Difficult Decisions

In situations where counselling is unsuccessful, then hospital procedures are used to decide on the withdrawal or the treatment for patients declining treatment against medical advice. One male physician participant reported:

*As the attending physician, it is up to you to decide. You have the refusal form, so if the patient refuses the treatment and after counselling them, you write that they have declined the treatment. It might take away a burden from a physician, who might be considered irresponsible. So, the patient also writes to confirm that they have declined the specific treatment*. (#13, male physician, hospital A)

In addition, one participant reported that the ICU department is developing standard operating procedures (SOPs) to guide healthcare professionals in handling morally challenging situations concerning the management of critical patients and prioritization of patients in admitting them to the ICU.

*We started with the SOPs for acute and aggressive management and we are still working on them in the final stages. After that, the next guideline will be the criteria for patients to be admitted here. However, cases like these will also need guidelines, determining who should speak (on behalf of the patient) ….* (#25, male nurse, hospital B)

#### Subtheme 5: Consulting Management and Legal Units

In some cases, participants reported having resolved the challenge of patient’s refusal of treatment by using coercion to treat them. However, such medical interventions are done by consulting hospital management and legal units.

*… We had a case where a patient, who was a Jehovah’s Witness, refused to receive a blood transfusion…. Both the patient and their family refused the blood transfusion. In this case, the patient was administered anaesthesia…. So, we decided to transfuse the patient without his consent or their family’s knowledge*. *The hospital management team was involved in making this decision*. (#12, male nurse, hospital A)

*There was a case of a mother who refused to undergo a necessary operation because she believed it would prevent her from having more children. But she could have lost her life in her condition, so we sedated her and performed the operation without her consent…. We involved the legal personnel at the hospital*. (#2, female nurse, hospital A)

### Theme 2: Awareness and the Status of CECs in Health Care Settings

The participants were asked if they are aware of CECs, and whether there are CECS in their hospital settings. The majority of the participants indicated they were not familiar with CECs.

Subsequently, the first author elucidated the key functions and responsibilities of CECs within healthcare contexts. Conversely, some participants indicated awareness of ethics committees primarily concerned with administering disciplinary actions against healthcare professionals in instances of misconduct.

*… in our hospital settings, ethics issues emerge when disciplinary incidents (like using abusive language while talking to patients and family members) occur and instances where healthcare professionals commit misconduct.* (#13, male physician, hospital A)

Another participant mentioned a clinical auditing committee* in the previous hospital.*

*…. I encountered a hospital with a clinical auditing committee responsible for both clinical matters and ethics*” (#11, female physician, hospital A)

She added that: *“having a clinical ethics committee in our hospital is very important. If established in our hospital, it will assist in handling increasingly severe moral dilemmas.”* (#11, female physician, hospital A)

### Theme 3: Perceived Key Needs for Establishing CECs in Healthcare Settings

After explaining the various functions of CECs by the first author, participants were asked to identify the key requirements for establishing CECs in Tanzania. They highlighted two main needs in their responses: (1) *ethics capacity building of healthcare professionals and community members* and (2) *the need for help in discussing moral challenges*.

#### Subtheme 1: Ethics Capacity Building of Healthcare Professionals and Community Members

Participants highlighted that if a CEC is established in their respective hospitals, it would assist in building the capacity of healthcare professionals and community members to address clinical ethical issues. Some participants said:

*We need a clinical ethics committee in our institution. If established in the hospital setting, ethics education will be offered to healthcare providers since some might not have received any ethics training.* (#12, male nurse, hospital A)

*The clinical ethics committee is essential, but it should not only focus on clinical practices but also build the capacity of community members about health matters. They can use the media … to create ethical awareness by specifying the ethical issues associated with healthcare delivery services and patients’ responsibilities and duties.* (#23, female nurse, hospital B)

#### Subtheme 2: The Need for Help in Discussing Moral Challenges

Participants expressed that if a CEC is established, it would offer a platform to discuss moral challenges in hospital settings. One of the female physicians explained a concrete case where a CEC would be used: “*We had a case where family members refused a blood transfusion, but the patient was unconscious, and family members made the decision, so we didn’t know if the patient refused or not. In such situations, the clinical ethics committee should assist healthcare professionals in deliberating ethical decisions*” **(#**18, female physician, hospital B).

Another participant mentioned that a CEC would offer a platform for moral deliberations in clinical practice: “*For healthcare workers, especially when we face challenges that lead to a point where we can’t make decisions, having a committee like this can help in discussing alternative courses of actions in collaboration with the family…*” (#19, male nurse, hospital B).

Moreover, participants reported that a CEC would be significant in assisting healthcare professionals to resolve moral challenges in clinical practice. Participants offered a variety of needs that could be addressed by implementing a CEC in hospital settings.

*They could … organize ethics sessions for us at the department level.* (#11, female physician, hospital A)

*We should have a committee because we currently address issues, but not as a committee. Although social welfare officers assist us, a committee would be essential*. (#17, female nurse, hospital B)

*We clinicians are often indoors and focused on clinical issues, so I believe it would be beneficial to have clinical ethics committee members engaging with us in our practice and assisting in addressing moral challenges. They could attend our operational meetings periodically or organise ethics sessions for us at the department level.* (#11, female physician, hospital A)

## Discussion

In this discussion section, we are reflecting upon the study findings and comparing them with previous studies on the subject matter.

### Reported Implicit Approaches for Handling Moral Challenges

Our findings indicate that health professionals in Tanzania use various ways to deal with moral challenges. The reported approaches can be categorized as implicit CES services in healthcare settings. Previous studies conducted in health institutions in higher-income settings make a distinction between implicit CES and explicit CES. On the one hand, implicit CES have been described as situations in which healthcare professionals do not frame the issues explicitly as being ethical or moral issues, or not using specific meetings or methods with an explicit focus on ethics or moral reasoning (Molewijk et al., 2015). In another study, implicit CES services have been described as both informal and formal structures in healthcare settings, such as individual consultations, group meetings, conversations with individual colleagues, education sessions, and procedures in which the ethical aspects of care are addressed indirectly (Dauwerse et al., 2014). On the other hand, explicit CES services have been described as institutionalized structures with a formal role regarding ethical issues in health care. These include CECs, individual ethics consultants, and MCDs (Dauwerse et al., 2014).

Quite a few participants mentioned they discussed moral issues and challenges in different meetings with co-workers. These meetings were not typically structurally designed to discuss moral challenges. Such ways have been referred to as implicit ways of dealing with moral challenges. Even though implicit CES services are used and often experienced as valuable and useful, participants recognised the need for more systematic and well-structured ways of dealing with moral challenges.

Some participants reported that they referred moral challenges and issues to their hospital social welfare units. Previous studies elsewhere present the role of social welfare units as hospital structures in handling moral challenges (Chitereka, 2010; Dako-Gyeke et al., 2018). Some of the reported key roles of social workers include providing the contextual focus necessary for client – and family-centred care; ensuring effective communication between patients and other health professionals by addressing possible conflicts/ or responding to distress; facilitating patient and family engagement in case or conflict management; as well as advocating for the interdisciplinary team within the healthcare institution to promote client’s and families’ decision-making (Chitereka, 2010; Dako-Gyeke et al., 2018). Based on the crucial role that social welfare units play in managing morally conflicting situations in Tanzanian healthcare settings, we recommend including a member from the social welfare unit in implementing CECs as explicit CES in Tanzanian healthcare settings.

Some participants mentioned handling moral challenges related to refusal of planned treatment based on religious and cultural beliefs through legal and management channels. In Tanzania, religious beliefs notably impact medical interventions, often guided by religious leaders and organizations (Sambaiga et al., 2019; Marten, 2022). The majority of Tanzanians consider religion to be an essential part of their lives, with Christianity (55.33%) and Islam (31.55%) being the predominant religions (Aid to the Church in Need (ACN) International, 2023). These religious and cultural perspectives on healthcare delivery are pertinent to clinical ethics discussions, which could benefit from explicit, systematic, and well-structured CES in hospital settings (Kuhumba et al., 2024).

In addition, managing the challenge related to refusal of treatment through hospital legal and management channels raises a moral concern in clinical practice. For example, on the one hand, a healthcare professional – physician, nurse, physician assistant or other professional has a duty of beneficence toward the patient. This implies an obligation to use her/his medical expertise to do good and to avoid harming a patient. On the other hand, the patient has a right to this skilled beneficence and to respect for his/her autonomous choices regarding what the healthcare professional does (Vaughn, 2017). Conflicts between respect for patient’s autonomy and healthcare professional’s duty of beneficence usually raises the issue of paternalism, which can be referred to as the overriding of a person’s actions or decision-making for his/her own good (Vaughn, 2017). Therefore, the implementation of systematic and structured CES services like CECs could provide a forum to discuss conflicts between ethical principles, religious and cultural values that influence the delivery of healthcare in Tanzania.

Based on the findings in this paper and the literature discussing various implicit ways of CES, we recommend implementing more explicit systematic and well-structured ways of addressing moral challenges and supporting healthcare professionals and healthcare institutions in dealing with these moral challenges. Explicit CES can be an important service in addition to implicit CES; implicit CES has an important and valuable role in supporting health care professionals, but also has some disadvantages such as a lack of ethics focus and clear conversation methods for moral deliberation. Besides, implicit CES provides an open, organic and narrative approach to the ethical dimension of care by evoking stories (Dauwerse et al., 2014). Implicit CES offers healthcare professionals the opportunity to discuss and integrate moral issues in their common practices in an organic and narrative way (Dauwerse et al., 2014, 106). Therefore, implicit CES activities can create a social basis for establishing explicit kinds of CES. Also, implicit CES activities could support the integration of explicit CES in hospital settings.

Given the presence of implicit CES activities in the hospitals studied, the study participants expressed a need for a more structured approach, particularly a CEC. Thus, a CEC could improve the quality of healthcare delivery by offering a platform to discuss moral issues occurring in clinical practice, assist in designing hospital policies and guidelines that are sensitive to ethical issues, as well as improve the moral and professional competency of healthcare practitioners through capacity-building programmes. The reason for integrating explicit CES is that the implicit CES lacks a clear structure and method to discuss moral challenges. Instead, explicit CES is more advantageous because it addresses the ethical dimensions of care in structural, professional and systematic ways (Dauwerse et al., 2014). Consequently, explicit CES could strengthen ethical capacities through learning from reported and deliberated moral cases and ethical issues at the organizational level such as resource allocation in equitable ways in the hospital settings.

### Lack of CECs in the Studied Hospitals

In this study, we found out that none of the study sites have CECS to handle morally challenging situations in clinical practice. Some studies based on Sub-Saharan report that in most countries, CECs have not been implemented in the healthcare system (Aboud et al., 2018; Moodley et al., 2020, 2021; Nanyonga et al., 2024). For example, a study by Moodley et al. indicates that the majority of participants were unfamiliar with CECs; instead, they conflated CECs with Research Ethics Committees (RECs). In addition, approximately 85.3% reported no formal CECs in their institutions (Moodley et al., 2020). Yet, 14.7% of participants reported that they had CECs in some of the hospitals (Moodley et al., 2020). Despite a few CECs being implemented in hospitals and health institutions, little has been discussed about their implementation process, common cases reported in CECs, and specific barriers and facilitators for establishing CECs in the Sub-Saharan context. Therefore, studies about established CES and CECs in hospitals around Africa are relevant for informing the implementation of CES and CECs in other countries, particularly low-middle income countries.

### Key Needs for Establishing a CEC

Once participants were informed about the roles and responsibilities of a CEC, they confirmed the need for implementing more systematic and structured modalities to address moral challenges. The participants indicated various key needs for establishing a CEC: ethics capacity building of healthcare professionals and community members, as well as the need for help in discussing moral challenges in a systematic way.

Studies conducted in the Sub-Saharan context have identified the need for establishing CESS services such as CECs (Aboud et al., 2018; Dzi et al., 2024; Moodley et al., 2020, 2021; Nanyonga et al., 2024). Some of these studies have emphasized that first there is a need for building expertise in clinical ethics and building expertise for CEC members who are usually not ethicists or clinical ethicists (Aboud et al., 2018; Moodley et al., 2021). Similarly, our study findings highlighted not only the need for capacity building for potential CEC members concerning clinical ethics and the methods of conducting ethical deliberations and reflections in CEC discussions and meetings, but also the need to educate community members about clinical ethics issues.

Therefore, based on the findings of this study, we suggest the implementation of explicit CES services, particularly CECs, in Tanzanian healthcare settings. One of their tasks could be enhancing the moral competencies of healthcare practitioners, especially in healthcare settings where healthcare professionals lack skills in clinical ethics. A previous study indicates that the majority of studied healthcare professionals reported having limited training in clinical ethics and some expressed that during their formal training as healthcare professionals, little consideration was given to ethics reflection on moral issues, challenges, and uncertainties occurring in clinical settings (Kuhumba et al., 2024). By focusing on capacity-building initiatives, such as clinical ethics training and education, healthcare professionals and support staff will be better equipped to identify and address moral issues and seek assistance from established clinical ethics support when necessary.

Although CECs can support Tanzanian healthcare professionals in addressing moral challenges, they can also serve other functions, such as educating staff through seminars and developing (policy) guidelines for practice. In this way, the establishment of a CEC will not only provide valuable and wanted help for healthcare professionals dealing with moral challenges, but it can also help to raise awareness about clinical ethics and educate healthcare professionals on how to deal with moral challenges in their practices (Magelssen et al., 2020a, 2020b). If healthcare professionals receive clinical ethics education, they might use a CEC more easily and more often. Moreover, CEC could assist in providing assistance involving ethics at the level of the institutional health system as opposed to the level of patient care (Doran et al., 2016). This role usually entails working through ethical issues involved in areas such as healthcare management, resource allocation, and quality improvement.

Additionally, by implementing CES, healthcare institutions should not only focus on establishing explicit CES services but also using existing implicit CES more appropriately. Implicit CES can serve as a stimulus to the implementation of explicit CES and may also enhance the integration of explicit CES in healthcare settings (Dauwerse et al., 2014). Therefore, in the studied hospitals implicit CES, including meetings, hospital guidelines, existing structures such as the social welfare units, and family involvement could serve as a social basis to implement explicit CES, particularly a CEC. Hence implicit CES and explicit CES could be integrated and strengthen each other in healthcare settings.

Globally, international organisations have emphasised the need to implement CES services in healthcare systems; for example, according to UNESCO’s Universal Declaration on Bioethics and Human Rights, ethics committees should be established to advise on ethical problems in clinical settings (Ten Have et al., 2011; Huriet, 2009). Global initiatives are underway to integrate CES services into healthcare systems. But WHO, for example, has so far, not issued any explicit guidance regarding clinical ethics and CES structures. The need for such guidance has been repeatedly expressed by various technical departments and the Global Network of WHO Collaborating Centers for Bioethics (Reis & Shamsi-Gooshki, 2023). Such global initiatives will advocate for clinical ethics support agendas by key stakeholders including healthcare institutions, ministries of health, and academic institutions.

The establishment and implementation of a CEC would require a responsive evaluation and a dialogical approach to adjust to the contextual needs and experiences. Responsive evaluation is characterized by dialogue (Abma, 2006) and a process-oriented approach (Abma et al., 2009). Responsive evaluation involves a structured way of setting up dialogical learning processes, by eliciting stories of participants, exchanging experiences in (homogenous and heterogeneous) groups, and drawing a normative conclusion for practice (Abma et al., 2009). Among the reasons for doing the responsive evaluation is for stakeholders to gain insight into the usefulness of a CEC, improve the implementation process, and create a shared sense of ownership of and responsibility for the use of the CEC (Hartman et al., 2019). Therefore, implementing a CEC requires competence, building networks and international collaborations to facilitate joint moral learning, thereby focusing on fostering the quality of care. We recommend collaborations between global North and global South institutions in implementing CECs in healthcare institutions, as well as establishing regional collaboration among African networks such as the African Clinical Ethics Network.

## Study Limitations

Our study contributes to the existing literature on addressing moral challenges in healthcare settings, with a particular focus on Tanzania. Additionally, it sheds light on the key needs for establishing CECs services in Tanzanian healthcare. However, the study has some limitations. Primarily, it focuses on higher-level healthcare settings, potentially overlooking the ways used by healthcare professionals in lower-level settings to manage moral challenges in their daily clinical practices. Moreover, we might not have covered various ways used by healthcare professionals in the lower settings while dealing with moral challenges in their daily clinical practices. Also, we might not have covered the specific needs for establishing CECs at the lower settings. Therefore, we recommend further studies that include lower-level healthcare settings to gain a comprehensive understanding of how moral challenges are handled across the whole Tanzanian healthcare system and to identify the key needs for establishing more structured and systematic approaches to address moral issues in these contexts.

Second, our study included only nurses and physicians as representatives of healthcare professionals. We recognize that other healthcare professionals, such as pharmacists, laboratory technicians, hospital administrators, legal staff, and social welfare personnel, also might encounter moral challenges daily as they contribute to patient care. Consequently, we did not capture their experiences in handling moral challenges, as well as their needs for establishing CES services in healthcare settings. We recommend conducting further studies that engage a broader range of professionals contributing to the provision of healthcare services to understand the modalities they use to address daily moral challenges and to identify their specific needs in establishing more structured and systematic CES services, particularly CECs, in hospital settings.

Finally, this study focuses on three hospital units/departments (ICU, Emergency, and Internal Medicine). The selection of the studied departments was based on discussions between the first author and several local healthcare professionals. We acknowledge that including other hospital departments and units might have yielded different results. Therefore, we recommend that future studies involve a wider range of departments to obtain a broader perspective on how moral challenges are addressed and to identify the key needs for establishing more structured and systematic CES services in Tanzanian healthcare settings.

## Conclusion

Study participants reported that only implicit ways of dealing with moral challenges are used in the three Tanzanian hospitals (such as regular meetings, family conferences, social welfare units, hospital procedures and guidelines, as well as consultations with legal and management units). Based on our findings in this study with respect to respondents’ perceived needs for CEC and related Sub-Saharan studies, we recommend the establishment of a CEC characterized by a multidisciplinary composition comprising members equipped with specialized training in clinical ethics and clinical ethics support. The implementation of CESs should be accompanied by a responsive evaluation process in order to continuously adjust the functioning of CECs to contextual needs and experiences and create shared ownership. Established CECs can assist in capacity building with respect to awareness and knowledge of clinical ethics; aid in developing policies and strategies in healthcare settings that are sensitive to ethical issues; provide assistance on dealing with moral challenges associated with healthcare management, resource allocation, and quality improvement; as well as provide a platform for healthcare professionals, patients and family members to engage in structured and constructive moral deliberations and reflections about morally challenging situations in healthcare.

We recommend future studies to engage with agencies at the top of the healthcare pyramid, in particular the Ministry of Health and President’s Office Regional Administration and Local Government, to understand their perspectives on implementing CECs in Tanzanian hospitals and how to develop regulations that both stimulate and support healthcare facilities to create a CEC. We also recommend future studies among patients and family members to capture their key needs and interests in making use of, and participating within, CECs.

## Supplementary Information

Below is the link to the electronic supplementary material.

Supplementary file1 (DOCX 22 KB)

Supplementary file1 (DOCX 41 KB)
