# Supplementary material for: Responding to Moral Challenges in Clinical Practice: A Qualitative Assessment of Clinical Ethics Support Needs at Three Tanzanian Hospitals
Source: HEC Forum. 2025 Apr 16;38(1):1–23. doi: 10.1007/s10730-025-09547-8 (PMC12876543; doi:10.1007/s10730-025-09547-8)
Supplement: Supplementary file 2 — Supplementary file1 (DOCX 41 KB) [file 10730_2025_9547_MOESM2_ESM.docx]

**Appendix 2: Indicating themes, sub-themes, codes and illustrative quotes**

| Themes | Sub-themes | Codes | Illustrative quotes |
| --- | --- | --- | --- |
| Reported approaches used to handle moral challenges in healthcare setting | **Meetings within a team of healthcare professionals**   - Meetings are not structurally framed to discuss moral challenges - Departmental meetings - Supervisory meeting - Mentorship programs - Team of healthcare professionals | - Moral challenges  - Moral dilemmas  - Clinical ethics  - Medical ethics  - Bioethics  - Ethics  - Departmental management meeting  - sharing experiences  - Resolving moral challenges  - Mentorship families  - educational sessions  - Family members  - Ethical deliberations  - Clinical practice  - Team of healthcare practitioners  - Misconducts  - Advisory role  - Team  - Decision  - Scarcity of medical resources | **Meetings are not structurally framed to discuss moral challenges**  *… it is mostly business as usual, so there is nothing new. I believe it is because we lack an effective system to track each case….* (#22, female nurse, Hospital B).  *the existing structure investigates misconduct more frequently. We don’t have a formal system that performs an advisory role on moral dilemmas and offers training on clinical ethics.* (#10, male physician, hospital A).  *In such meetings, experts in medical ethics, bioethics and ethics are not invited to assist in analyzing the case….*. (#9, female nurse, hospital A). |
|  |  |  | **Departmental meetings**  *We discuss moral challenges encountered in clinical settings*. *We learn from them, and how to address them if they arise again.* (#20, male nurse, hospital B).  *There is also a departmental management meeting every Wednesday…. So, we discuss these things quite extensively*. (#5, male nurse, hospital A).  *We educate and remind each other because some of the staff are newcomers, so they need to understand how to handle these challenges, and if they can't, they know who to seek guidance from.* (#17, female nurse, hospital B). |
|  |  |  | **Supervisory meeting**  *There is usually a morning report in these meetings, where the supervisor discusses everything related to clinical practice on that particular day. Any dilemma that has arisen is discussed during the meeting…. These meetings are conducted at the departmental level.* (#12, male nurse, hospital A). |
|  |  |  | **Mentorship programs**  *We hold weekly mentorship sessions to equip mostly junior medical staff with skills in handling moral distress in the ICU environment. Such sessions aim to strengthen healthcare practitioners and be morally resilient to ethical challenges encountered in the healthcare setting.* (#15, male physician, hospital A).  *Our department has a mechanism called mentorship families, where every junior doctor is assigned a senior specialist mentor. Your mentor is readily available, especially when you need to discuss moral challenges you encounter in clinical practice*. (#8, female physician, hospital A). |
|  |  |  | **Team of healthcare professionals**  *If the cancer patient is already on the ventilator, you cannot remove them just because another patient has arrived… In such situations, a team of healthcare professionals and a physician on duty decide who will benefit from the therapy based on the scarce medical resources available at the hospital unit.* (#10, male physician, hospital A). |
|  | **Making use of the Family conference**   - Family conference   ***Involvement of a social welfare unit*** | - Family conference  - Confusion  - ICU  - Uncertainties  - Autonomy  - Spiritual support services  - Family disagreements  - Education  - Refusal of treatment  - Family members  - Counselling  - Request to discharge against medical advice  - Team of healthcare practitioners  - Medical decisions  - Social welfare personnel  - Social and economic issues  - Counselling  - Family’s financial status  - Local government authorities  - Patient  - Treatment decline  - Decision-making | **Family conference**  *We have family conferences because when a patient is in the ICU, there is usually a lot of confusion and uncertainty about what has to be done. So, we involve them at every step. We call the family members and explain why the patient was brought to the ICU and the treatment plans. We also listen to their preferences, such as their desire for spiritual support services for their loved ones, which we accommodate.* (#19, male nurse, hospital B).  *The critical issue is usually to inform the relatives because the patient belongs to them, and they need to make decisions. It is their autonomy so a patient might need a specific treatment, but the family might disagree. Your role is to educate them. If they refuse, you document their refusal so that later, they don't turn around and accuse you of terminating the patient's treatment*. (#10, male physician, hospital A).  *My job is to educate and make them understand the treatment’s benefits and the consequences of declining the therapy*...” (#6, male physician, Hospital A).  **Counselling**  *We … provide counselling. It is challenging to withhold a patient from treatment just because relatives have requested it, so we make a significant effort to provide counselling. So far, counselling has been the primary approach to overcoming moral challenges related to the disagreement between family members and healthcare professionals on patient care.* (#34, female physician, hospital C).  *After the counselling process, a team of healthcare practitioners and parents decides to de-escalate or minimize care to the patient and allow the natural course to occur. It isn't easy to make such decisions, but we must make them… we involve parents in making such medical decisions.* (#15, male physician, hospital A) |
|  |  |  | **social welfare unit**  *We use social welfare personnel, especially in social and economic issues, especially when a person feels that medical treatment is too expensive and can’t afford it. The social welfare team provides counselling, and the patient continues to receive treatment while they assess the family’s ability to contribute to the medical expenses. They visit the patient’s home, interact with the local government authorities, and evaluate the situation to confirm that the patient is being treated under the exemption. ...* (#23, female nurse, hospital B).  *Sometimes, patients are required to go to the operating theatre, but their family refuses. The patient might be an adult but unable to make decisions for themselves. At the same time, we continue to persuade the family by involving social welfare personnel….* (#21, male nurse, hospital B). |
|  | **Use of hospital procedures and guidelines in handling morally difficult decisions**   - Hospital procedures - Guidelines - Designing standard operating procedures   **Consulting legal and management units**   - **Legal unit** - **Management unit** | - Physicians  - Decision making  - Patients  - Refusal of treatment/ decline of treatment  - Refusal form  - Standard operating procedures  - Acute and aggressive management    - Criteria for admission of patients  - Jehova’s witness  - Refusal of blood transfusion  - Patient  - Family  - Anaesthesia  - Consent  - Hospital management  - Decision making  - Sedation  - Operation / surgery | *As the attending physician, it is up to you to decide. You have the refusal form, so if the patient refuses the treatment and after counselling them, you write that they have declined the treatment. It might take away a burden from a physician, who might be considered irresponsible. So, the patient also writes to confirm that they have declined the specific treatment.* (#13, male physician, hospital A).  *We started with the SOPs for acute and aggressive management and we are still working on them in the final stages. After that, the next guideline will be the criteria for patients to be admitted here. However, cases like these will also need guidelines, determining who should speak (on behalf of the patient) ….* (#25, male nurse, hospital B) |
|  |  |  | **Consulting management unit**  *… We had a case where a patient, who was a Jehovah's Witness, refused to receive a blood transfusion…. Both the patient and their family refused the blood transfusion. In this case, the patient was administered anaesthesia…. So, we decided to transfuse the patient without his consent or their family's knowledge. The hospital management team was involved in making this decision.* (#12, male nurse, hospital A).  **Consulting legal unit**  *There was a case of a mother who refused to undergo a necessary operation because she believed it would prevent her from having more children. But she could have lost her life in her condition, so we sedated her and performed the operation without her consent…. We involved the legal personnel at the hospital.* (#2, female nurse, hospital A). |
| Awareness and the status of CECs in healthcare settings | - **Awareness of CECs** - **Lack of CECs** | - CECs  - Ethics issues  - Ethics  - Disciplinary incidents  - Healthcare professionals  - Misconduct  - Clinical auditing committee  - Moral dilemmas | *… in our hospital settings, ethics issues emerge when disciplinary incidents (like using abusive language while talking to patients and family members) occur and instances where healthcare professionals commit misconduct.* (#13, male physician, hospital A).  *…. I encountered a hospital with a clinical auditing committee responsible for both clinical matters and ethics*. (#11, female physician, hospital A).  *having a clinical ethics committee in our hospital is very important. If established in our hospital, it will assist in handling increasingly severe moral dilemmas.* (#11, female physician, hospital A). |
| Perceived key needs for establishing CECs in healthcare settings | **a) Ethics capacity building of healthcare professionals and community members**  **b) The need for help in discussing moral challenges** | - Capacity building  - Ethics education  - Ethics training  - Healthcare providers  - CEC  - Clinical practice  - Community members  - Health matters  - Media  - Ethical awareness  - Ethical issues  - Healthcare delivery services  - Patients’ responsibilities and duties  - Community members  - Health matters  - Media  - Ethical issues  - Ethical awareness  - Family members  - Blood transfusion  - Ethical deliberations  - Decision-making  - Ethics sessions  - Social welfare officers | **Ethics capacity building of healthcare professionals and community members**  *We need a clinical ethics committee in our institution. If established in the hospital setting, ethics education will be offered to healthcare providers since some might not have received any ethics training*. (#12, male nurse, hospital A).    *The clinical ethics committee is essential, but it should not only focus on clinical practices but also build the capacity of community members about health matters. They can use the media … to create ethical awareness by specifying the ethical issues associated with healthcare delivery services and patients’ responsibilities and duties*. (#23, female nurse, hospital B). |
|  |  |  | **The need for help in discussing moral challenges**  *We had a case where family members refused a blood transfusion, but the patient was unconscious, and family members made the decision, so we didn’t know if the patient refused or not. In such situations, the clinical ethics committee should assist healthcare professionals in deliberating ethical decisions.* (#18, female physician, hospital B).  *They could … organize ethics sessions for us at the department level.* (#11, female physician, hospital A).  *We should have a committee because we currently address issues, but not as a committee. Although social welfare officers assist us, a committee would be essential*. (#17, female nurse, hospital B).  *We clinicians are often indoors and focused on clinical issues, so I believe it would be beneficial to have clinical ethics committee members engaging with us in our practice and assisting in addressing moral challenges. They could attend our operational meetings periodically or organise ethics sessions for us at the department level.* (#11, female physician, hospital A). |
